# A nonsense variant in Rap Guanine Nucleotide Exchange Factor 5 (*RAPGEF5*) is associated with equine familial isolated hypoparathyroidism in Thoroughbred foals

**DOI:** 10.1371/journal.pgen.1009028

**Published:** 2020-09-28

**Authors:** Victor N. Rivas, K. Gary Magdesian, Sophia Fagan, Nathan M. Slovis, Daniela Luethy, Laura H. Javsicas, Brian G. Caserto, Andrew D. Miller, Anna R. Dahlgren, Janel Peterson, Erin N. Hales, Sichong Peng, Katherine D. Watson, Mustafa K. Khokha, Carrie J. Finno

**Affiliations:** 1 Department of Population Health and Reproduction, School of Veterinary Medicine, University of California-Davis, Davis, California, United States of America; 2 Department of Medicine and Epidemiology, School of Veterinary Medicine, University of California-Davis, Davis, California, United States of America; 3 Pediatric Genomics Discovery Program, Department of Pediatrics and Genetics, Yale School of Medicine, Yale University, New Haven, Connecticut, United States of America; 4 Hagyard Equine Medical Hospital, Lexington, Kentucky, United States of America; 5 Department of Clinical Studies–New Bolton Center, School of Veterinary Medicine, University of Pennsylvania, Philadelphia, Pennsylvania, United States of America; 6 Rhinebeck Equine L.L.P., Rhinebeck, New York, United States of America; 7 VetPath Services, Stone Ridge, NY, United States of America; 8 Department of Biomedical Sciences, Section of Anatomic Pathology, College of Veterinary Medicine, Cornell University, Ithaca, New York, United States of America; 9 Department of Anatomic Pathology, Veterinary Medical Teaching Hospital, University of California-Davis, Davis, California, United States of America; HudsonAlpha Institute for Biotechnology, UNITED STATES

## Abstract

Idiopathic hypocalcemia in Thoroughbred (TB) foals causes tetany and seizures and is invariably fatal. Based upon the similarity of this disease with human familial hypoparathyroidism and occurrence only in the TB breed, we conducted a genetic investigation on two affected TB foals. Familial hypoparathyroidism was identified, and pedigree analysis suggested an autosomal recessive (AR) mode of inheritance. We performed whole-genome sequencing of the two foals, their unaffected dams and four unaffected, unrelated TB horses. Both homozygosity mapping and an association analysis were used to prioritize potential genetic variants. Of the 2,808 variants that significantly associated with the phenotype using an AR mode of inheritance (*P*<0.02) and located within a region of homozygosity, 1,507 (54%) were located in a 9.7 Mb region on chr4 (44.9–54.6 Mb). Within this region, a nonsense variant (*RAPGEF5* c.2624C>A,p.Ser875*) was significantly associated with the hypoparathyroid phenotype (*P*_allelic_ = 0.008). Affected foals were homozygous for the variant, with two additional affected foals subsequently confirmed in 2019. Necropsies of all affected foals failed to identify any histologically normal parathyroid glands. Because the nonsense mutation in *RAPGEF5* was near the C-terminal end of the protein, the impact on protein function was unclear. Therefore, we tested the variant in our *Xenopus* overexpression model and demonstrated RAPGEF5 loss-of-function. This *RAPGEF5* variant represents the first genetic variant for hypoparathyroidism identified in any domestic animal species.

## Author summary

The Thoroughbred breeding industry in the United States produces a total impact of $6 billion. A lethal hypocalcemic syndrome was first described in young Thoroughbred horses in 1997. Affected foals suffer from seizures due to low blood calcium concentrations in the first few weeks of life. Our clinical evaluation of affected foals supported a diagnosis of primary hypoparathyroidism, and the disease appeared to be inherited as an autosomal recessive trait. Whole-genome sequencing of two affected foals identified a nonsense variant in *RAPGEF5*. Two additional affected foals were subsequently genotyped and also homozygous for the nonsense variant. Overexpression of the equine variant in frog embryos demonstrated loss-of-function. Genetic testing is now available to screen carrier Thoroughbreds and the disease was renamed equine isolated familial hypoparathyroidism (EIFH).

## Introduction

The Thoroughbred (TB) breeding industry in the United States produces a total impact of $6 billion [1]. Every year, approximately 20,000 TB foals are born in the United States and, of the ~7,000 sold as yearlings, the average price per yearling is approximately $77,000 [2]. Therefore, any genetic disease that results in loss of a Thoroughbred foal has tremendous economic consequences.

Five cases of refractory hypocalcemia in TB foals were described in 1997, with clinical signs including tetany, stiff gait, and hyperhidrosis. Four of the foals died and the fifth was euthanized based on a guarded prognosis [3]. These foals were hypocalcemic, typically hyperphosphatemic, and had low or normal parathyroid (PTH) concentrations despite hypocalcemia, suggesting hypoparathyroidism. Four of the foals had complete necropsy examinations. No parathyroid tissue could be identified grossly or histologically despite extensive examinations [3].

In humans, primary hypoparathyroidism results from iatrogenic causes (i.e. via surgical excision), autoimmune polyglandular syndrome or inherited causes. Familial isolated hypoparathyroidism includes autosomal recessive, autosomal dominant and X-linked recessive subtypes. Patients are hypocalcemic and often hyperphosphatemic, hypomagnesemic, and have inappropriately low or normal PTH concentrations. Symptoms and age of onset are extremely variable and dependent on the degree of hypocalcemia, ranging from asymptomatic to severely affected with recurrent seizures in infancy [4]. To date, genetic mutations have been identified in four genes. Pathogenic mutations in the *parathyroid hormone gene* (*PTH*) and *glial cells missing transcription factor 2 (GCM2*) have been associated with both autosomal dominant and recessive hypoparathyroidism. GCM2 is the only transcription factor with expression restricted to the parathyroid glands and is considered a secondary mechanism of calcium regulation [5]. Type 1 autosomal dominant hypocalcemia is primarily caused by mutations in the *calcium-sensing receptor gene* (*CASR*) [4], whereas mutations in the *G protein subunit alpha 11* gene (*GNA11*) lead to type 2 autosomal dominant hypocalcemia [6]. *CASR* encodes the calcium-sensing receptor (CaSR), responsible for increasing secretion of PTH during hypocalcemia. *GNA11* encodes for Gα11, a key mediator in CaSR signaling. Another gene, *transient receptor potential channel melastin 6* (*TRPM6*), has been reported to promote hypomagnesemia with secondary hypocalcemia in human patients [7].

To date, there have been no genetic mutations linked to primary hypoparathyroidism in any domestic animal species. We performed whole-genome sequencing on two affected TB foals with familial hypoparathyroidism to identify associated genetic variants within candidate or novel genes associated with the phenotype. The similar clinical presentation between familial isolated hypoparathyroidism in humans and equine idiopathic hypocalcemia led to the hypothesis that a coding variant within *PTH*, *GCM2*, *CASR*, *GNA11* or *TRPM6* would be associated with idiopathic hypocalcemia of TB foals. We definitively excluded these candidate genes in TB foals with familial hypoparathyroidism and instead identified a nonsense variant in *RAPGEF5* c.2624C>A,p.Ser875* that significantly associated with the phenotype. In order to test the impact of this equine variant on function, we used an overexpression model in *Xenopus* and found that the equine *RAPGEF5* variant was a loss-of-function allele.

## Results

### Pedigree analysis

**Fig 1:**
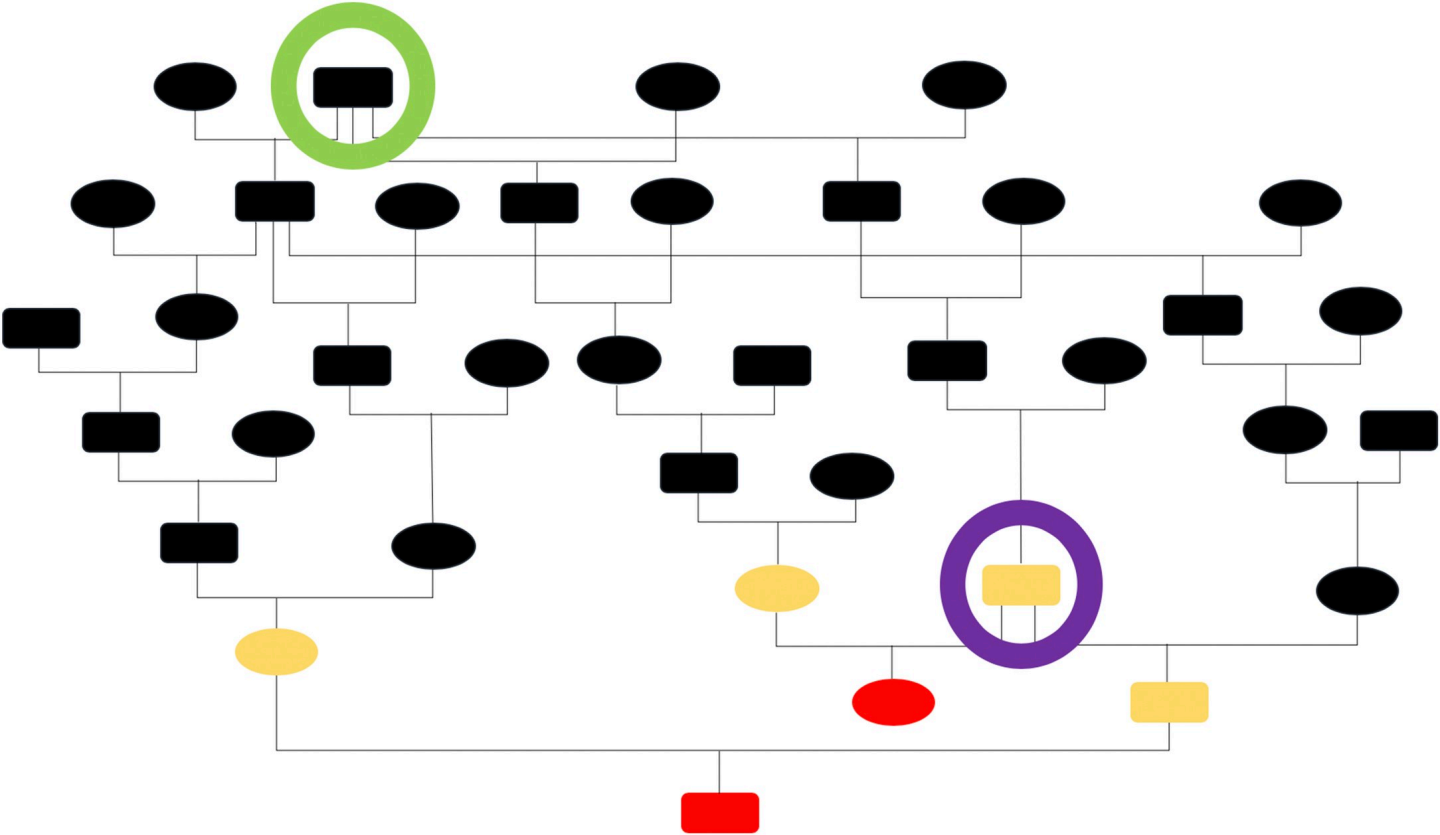
Pedigree of proband Thoroughbred foals affected with idiopathic hypocalcemia. A pedigree analysis was performed on the two proband affected foals (*Cases #1 and 2*) using Pedigraph (8). The sire of one affected foal was found to be the other affected foal’s grandsire (purple circle). Additionally, foals were related on the dam lines within six generations (green circle). Circles = females, squares = males, red = affected foals, yellow = unaffected horses, black = horses unavailable for evaluation.

The pedigrees of the two 2017 affected foals (*Cases #1 and 2*) were obtained and a pedigree analysis was performed using Pedigraph [8]. The sire of one affected foal was found to be the other affected foal’s grandsire. Additionally, foals were related on the dam lines within six generations. This pedigree analysis data suggested an autosomal recessive mode of inheritance for the disease (**Fig 1**). Subsequently, the two additional 2019 foals (*Cases #3 and 4*) were added to the pedigree, extending the lineage back ~12 generations (**S1 Fig**).

### Necropsy results

**Fig 2:**
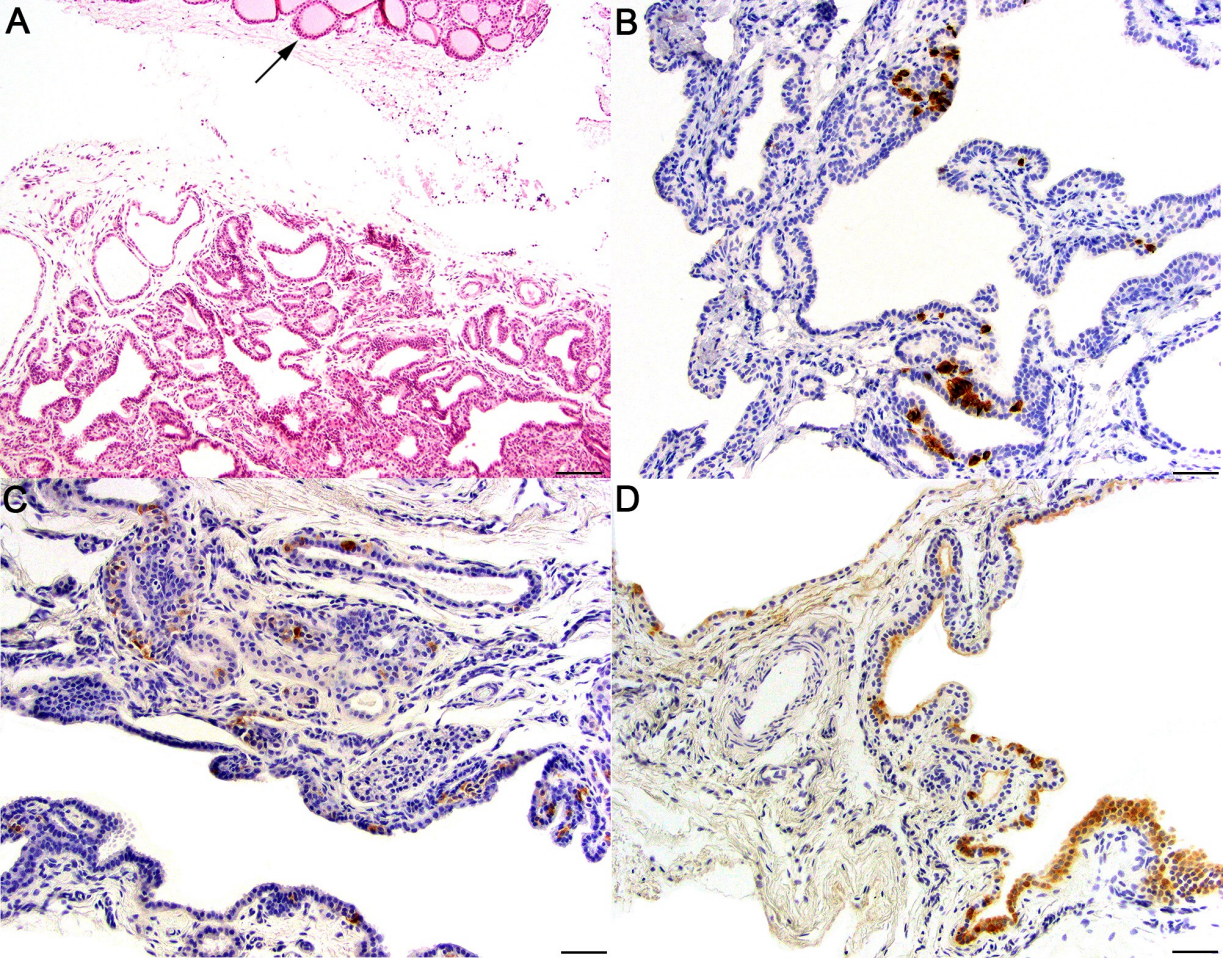
Necropsy of *Case #3*. Normal parathyroid was not observed histologically. However, a cystic structure was identified adjacent to the thyroid gland (A; arrow = thyroid gland, scale bar = 100 μm;), B-D This cystic structure had scattered immunoreactivity for (B) PTH, (C) calcitonin and (D) thyroglobulin (scale bar = 50 μm).

Clinicopathologic results supported a diagnosis of hypoparathyroidism in all foals (see *Methods*) and full necropsy examinations were performed. No gross or histologic lesions were present in the examined tissues from *Cases #1* and *2*. Examined tissues included the cerebrum, cerebellum, brainstem, pituitary, heart, liver, lung, kidney, adrenal gland, spleen, thymus, thyroid gland, stomach, small intestine, large colon and diaphragm. Despite extensive examination, the parathyroid glands could not be identified grossly or histologically in either case. In *Case #3*, multifocal myodegeneration and necrosis of skeletal muscle was identified. Normal parathyroid glands were not observed histologically. However, a cystic structure was identified adjacent to the thyroid gland (**Fig 2A**). This structure had scattered immunolabeling for PTH, calcitonin and thyroglobulin (**Fig 2B–2D**) and was interpreted as an embryologic remnant, possibly partially derived from a pharyngeal pouch.

In *Case #4*, pathological findings included moderate regionally extensive edema in the subcutis of the neck and shoulders; moderate, focal edema in the retroperitoneum; severe, subacute ulcerative gastritis; mild, acute, interstitial pneumonia; moderate, diffuse, sinusoidal neutrophilia in the liver; and small intestinal coccidiosis. No histologic abnormalities were found in the following tissues: brainstem, cerebellum, cerebrum, heart, gluteal muscle, kidney, thyroid gland, thymus, pancreas, pituitary, bladder, testes, adrenal gland, tongue, diaphragm, duodenum, jejunum, small colon, large intestine and eye. Parathyroid tissue was not grossly or histologically appreciable.

### Homozygosity mapping and exclusion of candidate genes

We performed whole-genome sequencing on the initial two 2017 foals (8.6 and 9.2x), their dams (8.9 and 9.1x) and two unrelated healthy TB (8.3 and 8.7x) at an average of 8.8x coverage. Whole-genome sequence data from an additional two unrelated healthy TB from the Functional Annotation of Animal Genomes (FAANG) archive was used as additional control horses (https://www.ebi.ac.uk/ena/data/view/PRJEB26698).Homozygosity mapping identified 23 regions, with the same homozygous allele shared between the two foals (average length 3.41 ± 2.54 Mb, range 0.43–10.8 Mb; **S1 Table**). Based on candidate gene location, all candidate genes (*PTH*, *GCM2*, *CASR*, *GNA11* and *TRPM6*) were excluded as these genes were not located in regions of homozygosity shared by the two affected foals. Therefore, these five candidate genes are unlikely to be implicated in familial hypoparathyroidism of TB foals.

### Whole-genome association study

A whole-genome association study was then performed to investigate the genetic cause of idiopathic hypocalcemia. Variants were filtered by the proposed mode of inheritance (homozygous alternate in the two affected foals and heterozygous in their dams), resulting in 66,112 variants genome-wide. These variants were further filtered using a Fisher’s allelic *P* value of <0.02, which would allow for one heterozygote in the control population (estimated Fisher’s exact allelic *P* value = 0.019). The resulting 4,973 variants were then compared to the identified regions of homozygosity to identify overlap.

Only six of the 38 regions of homozygosity contained variants that were significantly associated with the phenotype (**S1 and S2 Tables**). Within these six regions, 2,808 variants associated with the phenotype were included. Two of these regions were located on chr4, spanning 44940057–49262454 (one region) to 49326336–54598022 (second region), and included 1,507 (54%) of the associated, region-filtered, variants.

The 2,808 variants falling into regions of homozygosity were further filtered by effect (‘MODERATE’ and ‘HIGH’) using SnpEff [9] and screened in the NCBI SRA database (https://ncbi.nlm.nih.gov/subs/sra/). Only variants on chr4 and chr6 contained ‘MODERATE’ effects, which were identified in other breeds (**Table 1**). The only associated ‘HIGH’ effect nonsense variant was on chr4 (EquCab3.0 chr4:54108297) in exon 26 of *RAPGEF5* (c.2624C>A p.Ser875*) and was exclusive to TBs.

**Table 1:** ‘MODERATE’ and ‘HIGH’ genetic variants significantly associated with idiopathic hypocalcemia in Thoroughbred foals (*P*_allelic_<0.02). Associated variants in regions of homozygosity were screened across 123 horses within 12 breeds using the NCBI SRA database (https://ncbi.nlm.nih.gov/subs/sra/). AT = Akhal-Teke, FM = Franches-Montagnes, FD = Friesian dwarf, GWB = German Warmblood, HF = Haflinger, ICE = Icelandic, KWB = Koninklijk Warmblood, QH = Quarter horse, SH = Shetland pony, STBD = Standardbred, SWB = Swiss Warmblood, YH = Yakutian horse. At the time of screening, no other Thoroughbreds (TB) were identified in the NCBI SRA database.

CHROM	POS (EquCab3.0)	REF	ALT	SnpEff Putative Effect	*P*_allelic_	Non-TB breeds with variant
4	52229384	T	C	MODERATE (MACC1 c.1651A>G,p.Thr551Ala)	0.019	AT, FM, FD, GWB, KWB, QH, SH, STBD, SWB, YH
4	52229394	A	T	MODERATE (MACC1 c.1641T>A,p.Asn547Lys)	0.019	AT, FM, FD, GWB, KWB, QH, SH, STBD, SWB, YH
4	52229431	C	G	MODERATE (MACC1 c.1604G>C,p.Arg535Thr)	0.019	AT, FM, FD, GWB, KWB, QH, SH, STBD, SWB, YH
4	53619415	A	G	MODERATE (DNAH11 c.1036A>G,p.Thr346Ala)	0.019	FM, FD, GWB, KWB, QH, STBD
4	54108297	G	T	HIGH (*RAPGEF5* c.2624C>A,p.Ser875*)	0.008	None
6	15346206	T	G	MODERATE (COL4A3 c.626T>G,p.Leu209Arg)	0.019	AT, FM, FD, KWB, GWB, SWB, YH
6	15696790	C	G	MODERATE (LOC100062650 (c.1456G>C,p.Ala486Pro)	0.019	AT, FM, FD, GWB, HF, KWB, SWB
6	15924567	G	C	MODERATE (SPHKAP c.3787C>G,p.Arg1263Gly)	0.008	AT, FM, FD, GWB, KWB, YH
6	17133591	C	T	MODERATE (DNER c.389G>A,p.Gly130Glu)	0.019	FM, FD, HF, GWB, STBD, SWB
6	17133643	C	T	MODERATE (DNER c.337G>A,p.Gly113Ser)	0.008	FM, FD, HF, GWB, STBD, SWB, YH

### Variant validation via Sanger sequencing

DNA from both of the 2017 and 2019 foals, their dams and one of the foals’ sires was collected and Sanger sequencing performed for the putative *RAPGEF5* variant (c.2624C>A p.Ser875*). All four foals were homozygous for the variant and all dams and the one tested sire were heterozygous. DNA from the other foals’ sires were unattainable.

An additional 82 other available unaffected Thoroughbred horses were randomly selected from our database and genotyped. From the 82 horses, three were heterozygous for the nonsense variant and the remainder of the horses were homozygous wildtype (**S3 Table**). These three horses were unrelated to each other within three generations but could be connected back to the four affected foals within 3–6 generations. The allele frequency for the *RAPGEF5* variant was calculated for the 82 Thoroughbred horse population (*q* = 0.018).

### *RAPGEF5* conservation and expression

**Fig 3:**
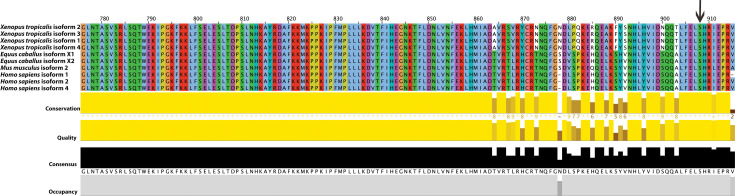
Alignment of protein sequences between *Xenopus tropicalis*, *Equus caballus*, *Mus musculus* and *Homo sapiens*. RAPGEF5 protein sequences from the two predicted equine NCBI proteins (isoform X1 = XP_023495120.1 and isoform X2 = XP_023495121.1), three human RefSeq proteins that extended to the 3’ end of the equine annotated protein (NP_036426.4 = isoform 1, NP_001354529 = isoform 2 and NP_001354531.1 = isoform 4), the only mouse RefSeq protein (NP_787126.3 = isoform 2) and four predicted *Xenopus tropicalis* (isoform 2 = XP_031759510.1, isoform 1 = XP_031759509.1, isoform 3 = XP_017950421.2 and isoform 4 = XP_031759511.1) proteins were aligned, demonstrating strong conservation even within alternate isoforms of RAPGEF5. The serine residue that is altered with *RAPGEF*5 c.2624C>A,p.Ser875* is highlighted with a black arrow toward the end of the sequence alignment.

The amino acid altered by the associated nonsense variant in *RAPGEF5* c.2624C>Ap.Ser875*, and 6/7 of the remaining amino acids in RAPGEF5, are 100% conserved across 100 vertebrate species (https://genome.ucsc.edu/; **Fig 3**). mRNA alignment of equine isoform X1 predicted *RAPGEF5* (XM_023639352.1) to equine isoform X2 (XM_023639353.1) and five validated human *RAPGEF5* mRNA sequences was performed using BLAST [10]. Only human transcript variants 1, 4 and 2 extended to the 3’ end of equine *RAPGEF5*, where the nonsense variant was located (**S4 Table**). RAPGEF5 protein sequences from the two resulting predicted equine NCBI proteins (isoform X1; XP_023495120.1; 882 aa and isoform X2; XP_023495121.1; 881 aa), three human RefSeq proteins that extended to the 3’ end of the equine annotated protein (NP_036426.4, NP_001354529, and NP_001354531.1), the only mouse RefSeq protein (NP_787126.3) and four predicted *Xenopus tropicalis* proteins (XP_031759510.1, XP_031759509.1, XP_017950421.2 and XP_031759511.1) were aligned, demonstrating strong conservation even within alternate isoforms of *RAPGEF5* (**Fig 3**). Of the two predicted equine proteins (isoform X1; XP_023495120.1 coded by XM_023639352.1 and isoform X2; XP_023495121.1 coded by XM_023639353.1), the only difference is an extra glutamine at position p.332 in isoform 1. Therefore, depending on the transcript model used, the *RAPGEF5* nonsense variant is either annotated as c.2624C>A,p.Ser875* (isoform X1) or c.2621C>A,p.Ser874* (isoform X2). When comparing alignments to human, mouse and *Xenopus*, however, the predicted isoform X1 containing the glutamine at p.332 is more likely based on conservation (**S2 Fig**). Therefore, we have annotated this nonsense variant based on the X1 isoform (*RAPGEF*5 c.2624C>A,p.Ser875*).

**Fig 4:**
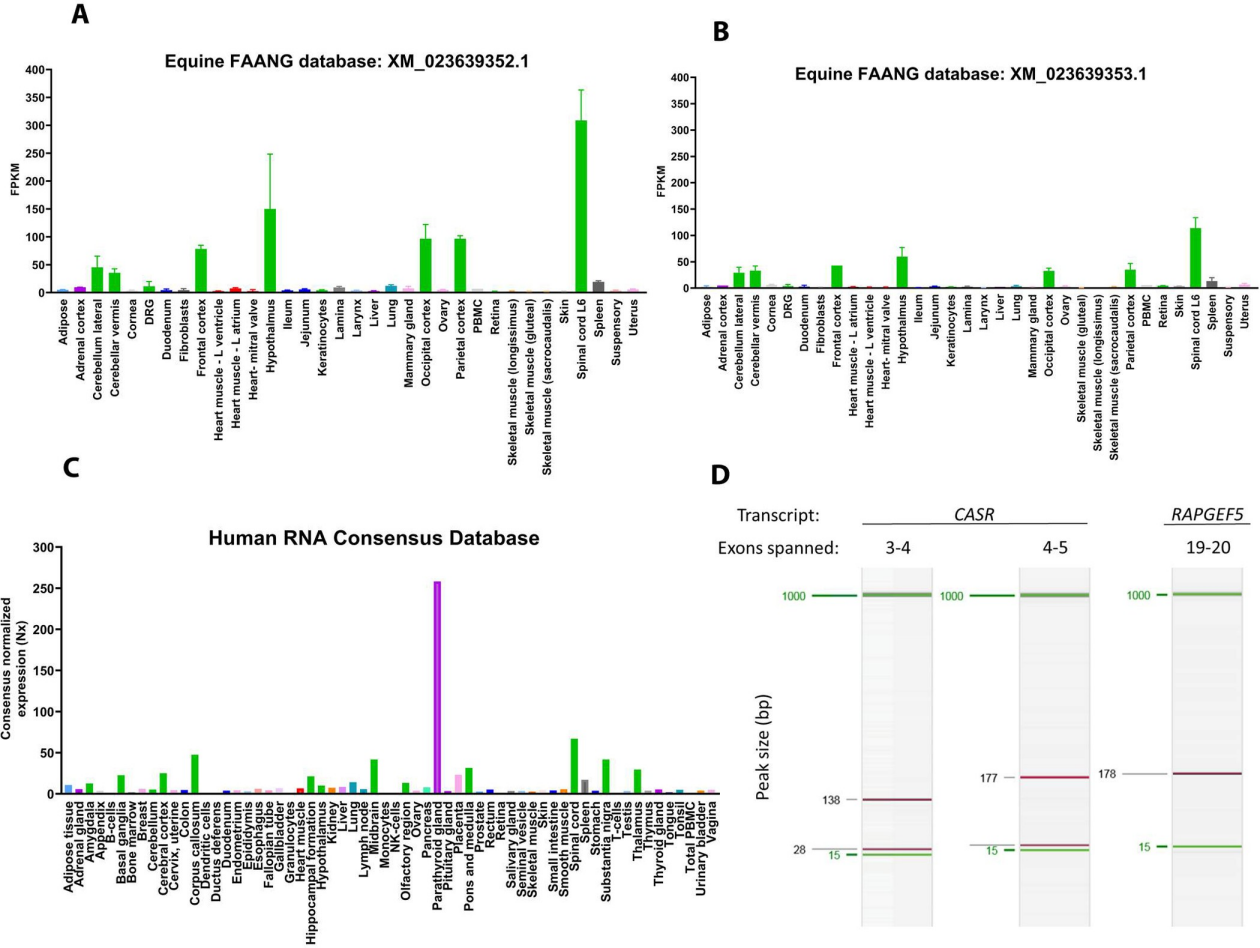
*RAPGEF5* transcript expression across tissues in horse (A, B) and human (C). In 45 equine tissues with publicly available RNA-sequencing data from the FAANG initiative, the two NCBI annotated equine transcripts of *RAPGEF5* (A) XM_023639352.1 and (B) XM_023639353.1 were most highly expressed in brain and spinal cord (green; median and 95% confidence interval graphed for n = 2 horses). Parathyroid tissue was not included in this biobank. (C) In humans, *RAPGEF5* transcript expression was enriched in the nervous system (green) and endocrine tissues (purple). Parathyroid gland had the highest RNA expression of *RAPGEF5*. FPKM = fragments per kilobase of transcript per million mapped reads, Nx = consensus normalized expression. (D) *RAPGEF5* is expressed in parathyroid tissue from a healthy control foal (primer sets spanning exons 19–20; expected product size 178 bp). Two primer sets, spanning exons 3–4 and 4–5 from *CASR*, were used as an internal control to confirm parathyroid tissue.

In 45 equine tissues with publicly available RNA-sequencing data from a Functional Annotation of Animal Genomes (FAANG) initiative (https://www.ebi.ac.uk/ena/data/view/ERA1487553) [11], the two NCBI annotated equine transcripts (https://www.ncbi.nlm.nih.gov/assembly/GCF_002863925.1/) of *RAPGEF5* (XM_023639352.1 and XM_023639353.1) were most highly expressed in brain and spinal cord (**Fig 4A and 4B,** respectively). As parathyroid tissue was not included in this database, the human protein atlas was used to evaluate parathyroid tissue expression of RAPGEF5 (https://www.proteinatlas.org/). RAPGEF5 protein expression was ubiquitous; however, transcript expression was enriched in brain (consensus normalized expression (Nx) 20–40, spinal cord (Nx = 67)) and within endocrine tissues. Of all tissues, parathyroid gland had the highest RNA expression of *RAPGEF5* (Nx = 258) (**Fig 4C**). While parathyroid tissue was not identifiable in any affected foals, tissue was collected from a healthy control foal and *RAPGEF5* expression demonstrated (**Fig 4D**).

### Protein structure

RAPGEF5 structure was searched using RCSB Protein Data Bank (http://www.rcsb.org/) [12]. Using the full protein feature view, a phosphorylation site was identified at the amino acid residue that is mutated in these foals (p.Ser573) within the Ras-GEF motif (**S3 Fig**). This phosphorylation site had been identified in T lymphocytes using mass spectrometry [13].

### *RAPGEF5* c.2624C>A p.Ser875* leads to loss-of-function of the coded protein

**Fig 5:**
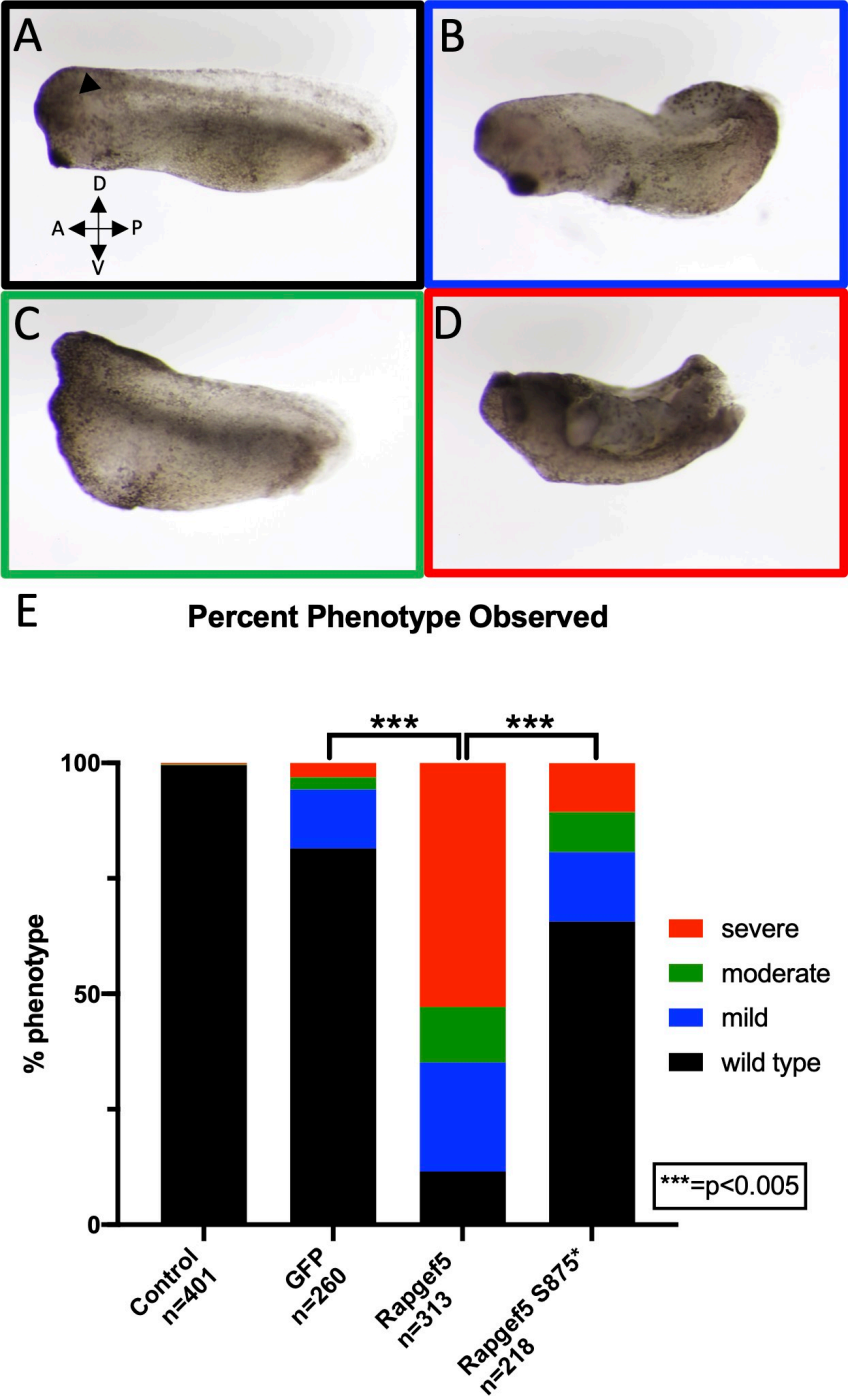
Overexpression model in *Xenopus* identifies the equine *RAPGEF5* variant as a loss-of-function allele. Overexpression of equine *RAPGEF5* mRNA affects embryonic development in *Xenopus tropicalis*; however, the equine S875* *RAPGEF5* variant has a dramatically diminished effect. (A) Normal development in uninjected control *X*. *tropicalis* embryos. All embryos are lateral views with anterior to the left and dorsal to the top. (B) Mildly affected development. Embryos display compromised elongation along anterior-posterior axis and a delayed formation of eyes and tail. (C) Moderately affected development. Most notably in this category embryos fail to form head structures (D) Severely affected development. Embryos display incomplete blastopore closure, incomplete neurulation, compromised elongation along anterior-posterior axis, and absence of distinguishable head structures. (E) Quantification of the phenotypes of the uninjected, equine *RAPGEF5* mRNA injected, and equine S875* *RAPGEF5* mRNA injected embryos classified as wildtype, mild, moderate, and severe at stage 28. Data is a compilation of three independent experiments. (*** p<0.0005). A = anterior, P = Posterior, D = Dorsal, Black triangle = filled arrowhead points to location of eye.

While genetic and expression analysis strongly suggested the association of p.Ser875* allele with idiopathic hypocalcemia in affected foals, the nonsense mutation lies very near the C-terminal end of the protein. Therefore, the impact of such a truncation on protein function was unclear. To test this, we overexpressed the equine *RAPGEF5* in *Xenopus tropicalis*. We previously showed that RAPGEF5 affects Wnt signaling and that overexpression of human RAPGEF5 in *Xenopus* can dramatically alter embryonic development [14]. We microinjected the wildtype equine *RAPGEF5* and the equine variant *RAPGEF5* and compared embryonic development to uninjected control embryos. While uninjected control embryos had no developmental phenotype (0%), embryos injected with GFP mRNA had a slight developmental defect (13% mild and 6% moderate/severe). In contrast, overexpression of wildtype equine RAPGEF5 mRNA resulted in a significant increase in severe (53%) developmental defects in stage 28 embryos with 12% having a moderate phenotype and 24% mild. Of note, these phenotypes are consistent with the known activity of RAPGEF5, namely activation of Wnt signaling (for example, the loss of head structures in **Fig 5C**; [14, 15]). Only 12% of embryos injected with wildtype equine RAPGEF5 mRNA appeared normal (**Fig 5;** *P* <0.005). However, overexpression of the equine S875* *RAPGEF5* variant produced predominantly wildtype embryos (66%), with the remainder of the embryos having a mild (15%), moderate (9%), or severe (10%) phenotype. The severity of these embryonic phenotypes appeared slightly more than GFP injected embryos but dramatically less than wildtype RAPGEF5 mRNA injected embryos (**Fig 5E**). From these studies, we conclude that the p.Ser875* variant is a loss-of-function allele.

## Discussion

Similarities between familial isolated hypoparathyroidism in humans and the refractory hypocalcemia observed in Thoroughbred foals led to the hypothesis that genetic variants in *PTH*, *GCM2*, *CASR*, *GNA11* or *TRPM6* may be responsible for the disease in the horse. Variants within these genes were not located within regions of homozygosity, thereby excluding these as likely candidates. We then pursued a whole-genome association study, resulting in the identification of a nonsense mutation in *RAPGEF5* (c.2624C>A p.Ser875*). The association of this variant with the equine familial hypoparathyroid phenotype was strengthened by its deep conservation across species, highest transcript expression in parathyroid tissue, confirmed association in two additionally affected foals in 2019 and by functional studies demonstrating a loss-of-function with this nonsense variant in our animal model. This genetic variant is therefore the first in any domestic animal species to be associated with primary hypoparathyroidism, and *RAPGEF5* should be considered an additional candidate gene for primary hypoparathyroidism in humans.

The equine variant is located in exon 26 of *RAPGEF5*, which is the last annotated exon in the horse. RAPGEF5 regulates nuclear translocation of β-catenin through activation of nuclear GTPases [14]. Beta-catenin plays a central role in directing several developmental processes through Wnt signaling. This interplay is critical during the formation of body regions in the early embryo. A link between the Wnt/β-catenin pathway and calcium signaling through PTH has been most well studied in bone. PTH stimulates the Wnt/β-catenin in mouse osteoblasts, leading to cell differentiation and bone formation [16]. The PTH receptor can also activate the β-catenin pathway directly by recruiting the adapter protein Dishevelled, independent of Wnt [17]. Another association with calcium-sensing receptors and Wnt/β-catenin signaling has been reported in the colon, where knocking out CaSR increases Wnt/β-catenin signaling [18]. The serine residue that is mutated in our foals correlates with a putative phosphorylation site in RAPGEF5 within the Ras-GEF motif at the C-terminus (**S3 Fig**). Indeed, our overexpression assay in *Xenopus* clearly demonstrates that the equine *RAPGEF5* variant cannot alter embryonic development nearly as effectively as the wildtype allele, indicating that the variant allele has lost its function.

The mechanism by which RAPGEF5 loss-of-function leads to hypoparathyroidism remains uncertain. Animal models to assess the function of RAPGEF5 are limited. Zebrafish with point mutations leading to premature stop codons exist but have not been phenotyped [19]. There is currently no published data on Rapgef5 mouse models. Despite expression of *RAPGEF5* in nervous tissue (**Fig 4A and 4B**), the clinical signs in our affected foals of seizures were attributed to the severe hypocalcemia and were prevented with calcium supplementation. Therefore, there did not appear to be a primary neurologic phenotype. Extensive necropsy examinations in our affected foals did not identify any parathyroid tissue. While parathyroid tissue can be difficult to locate in healthy foals, we used immunohistochemistry in one foal to optimize our evaluation. Normal parathyroid tissue was not identified; however, a cystic structure adjacent to the thyroid, which had scattered immunoreactivity for PTH, calcitonin and thyroglobin, was observed (**Fig 2**). Based on the immunoreactivity, it is unclear if this remnant could be from the first and second pharyngeal pouches that develop into thyroid, or from the third and fourth pharyngeal pouches that develop into parathyroid. Thus, RAPGEF5 may play a role in derivation of the parathyroid gland, with the loss-of-function nonsense allele leading to aberrant development.

While expression of RAPGEF5 in human parathyroid tissue is evident (**Fig 4C**), these public datasets do not specify which transcript of RAPGEF5 is primarily expressed in this endocrine tissue. Alignment of our full-length cDNA probes to human validated RAPGEF5 isoforms conferred the highest coverage in human transcript variant 1 (NM_012294.5; **S4 Table**). Of the 13 transcripts predicted by Ensembl, the only NCBI RefSeq match is to NM_012294.5 (RAPGEF5-213 or ENST00000665637.1). Therefore, our equine cDNA probe included the conserved region found in the well-validated isoform of human RAPGEF5.

It is notable that, while three of our cases were young (16 h-4 d of age), *Case #4* did not present with severe hypocalcemia until 30 d of age. This colt was only mildly hypocalcemic at the initial examination (1 h of age; total calcium 10.7 mg/dL). This wide age distribution has been previously reported, with affected foals ranging from 4–35 d of age [3]. Foals consume milk as neonates, which is high in calcium. As daily calcium supplementation prevents these foals from demonstrating rigidity and seizures, the amount of calcium in the diet likely determines disease onset and progression. We therefore speculate that the amount of calcium ingested by foals may vary based on dam milk calcium concentration and amount of milk ingested.

Limitations of this study include whole-genome sequencing of only two affected foals. The 2019 foals were genotyped directly for the *RAPGEF5* nonsense variant rather than performing whole-genome sequencing since they were not identified until after the candidate mutation was discovered. This study demonstrates the potential for whole-genome sequencing association studies using small numbers of affected animals when the mode of inheritance is known, and variants are predicted to have a moderate to high effect. An unbiased allele frequency study for this variant was also not performed in the TB breed. Lastly, the role of RAPGEF5 in parathyroid development requires further investigation.

In conclusion, we have identified a nonsense mutation in *RAPGEF5* leading to hypoparathyroidism in neonatal TB foals. We further suggest renaming this disease to equine familial isolated hypoparathyroidism (EFIH). Due to the high economic value of many TB foals, genetic testing should be pursued to prevent breeding of carriers that produce affected foals.

## Materials and methods

### Animals

#### Ethics statement

For equine studies, all procedures were approved by the University of California-Davis Institutional Animal Care and Use Committee (protocol #20751) and carried out in accordance with guidelines and regulations. Written owners’ consent was obtained for all sample collections. For *Xenopus* studies, *Xenopus tropicalis* were housed and cared for in the aquatics facility following Yale University Institutional Animal Care and Use Committee (protocol # 2018–11035) and all methods carried out in accordance with this protocol. Embryos were produced and raised as previously described [14].

#### 2017 cases

Two foals diagnosed with idiopathic hypocalcemia were initially used in this study. In the spring of 2017, a 16-hour old TB filly (*Case #1*) was admitted to the Hagyard’s Equine Medical Institute (Lexington, KY) with stiff gait, tetanic weakness, fevers (105°F; reference range 99–101°F), and excessive recumbency. At admission on this first visit, the foal was bright and alert, but febrile (102°F). Initial blood work revealed hypocalcemia (total calcium 7.0 mg/dL; ref: 10.8–12.8 mg/dL), hypomagnesemia (total magnesium 1.1 mg/dL; ref: 2.0–3.2 mg/dL), hyperphosphatemia (8.4 mg/dL; ref 1.3–6 mg/dL) and increased liver enzymes. Despite the profound hypocalcemia, serum PTH concentration was normal at 1.20 pmol/L (ref: 0.60–11.0 pmol/L). The filly was maintained on high doses of intravenous fluids containing calcium gluconate. Subsequent blood work revealed refractory hypocalcemia (total calcium 9.8 mg/dL; ionized calcium of 4.7 mg/dL [ref: 5.0–7.0 md/dL]) and a normal phosphorus of 5.7 mg/dL (ref: 4.1–6.4 mg/dL). The foal was discharged on an oral calcium carbonate supplementation and periodic blood work re-evaluations. One month after the first visit (May 2017), the foal was re-admitted after identifying recurrent hypocalcemia (total calcium 7.9 mg/dL) three days prior. An intravenous catheter was placed for the administration of fluid therapy with calcium and magnesium. Hours after supplementation, blood work evaluations revealed resolved hypomagnesemia, but refractory hypocalcemia. The filly was discharged on calcium carbonate powder orally three times per day and IV calcitriol twice per week. DNA samples and a complete pedigree from this foal and its clinically healthy dam were obtained prior to the foal dying after one missed supplementation. A full postmortem examination was performed.

The second case was a 2.5-day old colt (*Case #2*) that was presented to the UC Davis Veterinary Medicine Teaching Hospital (Davis, CA) in February of 2017 with stiff gait, muscle rigidity, and an inability to stand. The gestation length of the colt was normal at 351 days. Normal delivery was observed. The foal was acting and behaving normally until a few hours postpartum, when muscle stiffness was first observed. The foal was febrile (103.6°F), tachycardic and displayed severe muscle rigidity, that progressed to the foal being unable to stand without assistance. Blood work revealed hypocalcemia (total calcium 6.4 mg/dL; ionized calcium 3.25 mg/dL), hypomagnesemia (total magnesium 1.4 mg/dL; ionized hypomagnesemia 0.58 mg/dL [ref: 1.02–1.7 mg/dL]), hyperphosphatemia (8.4 mg/dL [ref: 1.3–6.0 mg/dL]) and an inappropriately normal PTH concentration (0.9 pmol/L). Serum vitamin D concentration was normal at 13 nmol/L (ref: 11–24 nmol/L). These findings were consistent with hypoparathyroidism. The foal was administered intravenous calcium, magnesium, dextrose and diazepam for muscle relaxation and seizure prevention. The foal was transitioned to oral calcium, magnesium and vitamin D after a 48-hour period of response to IV supplementation. The foal was discharged on a recommended medication plan of calcium carbonate, magnesium and vitamin D supplementation after obtaining DNA samples and a complete pedigree from this foal and its clinically healthy dam. The owner reported that the foal was experiencing recurrent seizures one month after discharge. The colt underwent a full necropsy evaluation.

#### Control horses

Whole blood samples were collected from two additional TBs (11-year old gelding and 8-year old mare) from UC Davis’s Center for Equine Health research herd. Both horses were clinically healthy and unrelated to the affected foals and their dams. Additional unaffected TB samples were from two mares (4 and 5-years old) that were sequenced for the Functional Annotation of Animal Genomes (FAANG) initiative, as previously described [11], with whole-genome data publicly available at the European Nucleotide Archive (https://www.ebi.ac.uk/ena/data/view/PRJEB26698).

### Blood DNA extraction

Genomic DNA was isolated from whole blood samples according to the WIZARD Blood DNA Extraction Kit protocol (Promega, Madison, WI). Sample DNA concentrations were measured using QIAxpert (QIAGEN, Hilden, Germany) and diluted to 20 ng/μl.

### Whole-genome sequencing

Genomic DNA from the six horses were sequenced on the Illumina HiSeq2500 at approximately 9X coverage. Whole genome sequences were deposited in the NCBI Sequence Read Archive (https://ncbi.nlm.nih.gov/subs/sra/) (PRJNA601992). Fastq files were obtained from the FAANG archive (https://www.ebi.ac.uk/ena/data/view/PRJEB26698) and trimmed for quality. Reads were mapped to the EquCab3.0 equine reference sequence [20] using BWA for Illumina mapping program [21]. Mapping quality was assessed using Samtools Flagstat [22]. SNP, INDEL discovery and genotyping across all samples was performed for the discovery of a variant using FreeBayes [23].

### Homozygosity mapping

SNPSift [24] was first used to filter the resulting .vcf file by quality, using a variant Phred threshold of 30 (Q≥30) and then pruned for strong local linkage disequilibrium using PLINK (--indep-pairwise 50 50 0.2) [25], leaving 437,062 variants for homozygosity mapping. Homozygosity mapping was performed in plink using a 50 kb sliding window and allowing for n = 3 heterozygotes in each window (i.e. two dams and one unaffected horse). Overlapping segments were identified using–homozyg-group and further filtered by regions of shared homozygous alleles in only the two affected foals.

### Candidate gene evaluation

Candidate gene regions (*PTH*, *GCM2*, *CASR*, *GNA11* and *TRPM6*) were compared to regions of homozygosity using EquCab3.0 coordinates for each candidate gene and allowing for 1 kb up- and down-stream from the annotated start and stop codons, using the Ensembl annotation for EquCab3.0 (http://m.ensembl.org/Equus_caballus/Info/Annotation).

### Whole-genome association study

A whole-genome association study was performed once candidate gene regions were excluded. SNPSift [24] was first used to filter the resulting .vcf file by quality, using a variant Phred threshold of 30 (Q≥30). Next, variants were filtered using SNPSift [24], with the assumption that the disease has an autosomal recessive mode of inheritance. Variants were filtered by requiring the two affected foals to be homozygous variant (isHom & isVariant) and the two unaffected dams to be heterozygotes (isHet). This resulted in 66,112 variants that were then assigned case/control status using SNPSift caseControl, followed by filtering by an allelic *P* value of <0.02, allowing for one heterozygote in the control horse population. In addition to assessing significantly associated variants called with freebayes, raw bam files were visually inspected using Integrative Genome Viewer [26] in the candidate gene regions and regions of homozygosity for any structural variants, including duplications, inversions and large deletions or insertions. After regions were compared to regions of homozygosity, SNPEff was then used to predict the effect on protein function of the alternate variants within these candidate regions, classifying variants as having ‘HIGH’, ‘MODERATE’, ‘LOW’ or ‘MODIFIER’ putative effects [24]. Variants with predicted ‘HIGH’ or ‘MODERATE’ effects were then screened in the publicly available database of equine whole-genome sequences using NCBI’s Sequence Read Archive (SRA) database (https://www.ncbi.nlm.nih.gov/sra) and excluded if found in breeds other than the TB.

### Sanger sequencing validation

To confirm the putative variants identified on whole-genome sequencing, primers were designed using Primer3Plus software [27] and DNA oligonucleotides synthesized by Invitrogen technologies (F 5’AACGTCTCCCTGTTTCATGC3’, R 5’GCTTGCTGTGTGTCTGTGCT3’) (Thermo Fisher Scientific, Waltham, MA). Amplification of products was performed using end-point PCR and visualized with the QIAxcel Advanced System (QIAGEN) and the QIAxcel DNA Screening Kit (QIAGEN). The 20-μl PCR reactions comprised 2 U of Hot-start TAQ and 2.0 μl of 10x buffer (Applied Biosystems), 0.25 mM of dNTPs (Thermo Fisher), 0.5 mM of both forward and reverse primers (Invitrogen Life Technologies) and 1 μl of 20 ng of genomic DNA. Standard PCR conditions were performed as follows: 95°C for 10 min, 35 cycles of 95°C denaturation for 30 s, 60°C annealing for 1 min, 72°C extension for 1 min and a final extension at 72°C for 10 min. PCR products were purified using the ExoSAP-IT PCR Product Cleanup Kit (Affymetrix). Sanger sequencing was performed using ABI 3730 Capillary Electrophoresis genetic analyzers (Applied Biosystems). Resulting sequences were aligned to EquCab3.0 (http://www.ncbi.nlm.nih.gov/genome/145) and analyzed with SEQUENCER software (Gene Codes Corp.). The two 2017 affected foals, their dams and the initial n = 4 control horses were genotyped. An additional n = 82 other unaffected Thoroughbred samples from our laboratory were genotyped for the *RAPGEF5* nonsense variant (**S1 Table**).

### *RAPGEF5* conservation and expression

Scores were determined for each orthologous human variant using the 100-vertebrate score by phastCons (https://genome.ucsc.edu/) since conservation scores are not available in the EquCab3.0 genome browser within UCSC (https://genome.ucsc.edu/). *RAPGEF5* expression was evaluated in tissues available from the equine FAANG initiative (https://www.ebi.ac.uk/ena/data/view/ERA1487553) [11]. Raw reads were mapped to EquCab3.0 using STAR [28], and fragments per kilobase of transcript per million mapped read (FPKM) determined using salmon [29] per horse per tissue. The equine FAANG dataset does not include parathyroid tissue. Therefore, the human protein atlas (https://www.proteinatlas.org/) was also used to identify parathyroid RNA and protein expression in humans.

Parathyroid tissue was collected from a two-week old Quarter horse foal that was euthanized for neurologic disease unrelated to hypoparathyroidism. Total RNA was extracted using TRIzol reagent (Thermofisher, Wilmington, DE, USA). RNA was then washed and eluted on columns (Direct-zol RNA MiniPrep Plus, Zymo, Irvine, CA), treated with TURBO DNase (Thermofisher, Wilmington, DE, USA) and converted to cDNA (SuperScript III, Thermofisher) per the manufacturer’s instructions. Primers were designed in Primer3plus [27] and selected only if pairs spanned at least one intron. Two primer sets spanning exons 3–4 (F 5’CAATGGTTCCTGTGCAGACA3’ and R 5’TCCCCACTAGGTCACCACAT3’) and exons 4–5 (F 5’GGCTCCATCGTGTTTAAGGA3’ and R 5’CCCCTCAATGATTCCTTTCC3’) of *CASR* were used as internal controls to validate parathyroid tissue. Primers spanning exons 19–20 of RAPGEF5 were used (F 5’CAGCAAAGGTCGATGAGGAT3’ and R 5’TCATTGCATCTCTGGAGCAG3’). PCR Reactions were performed in a 10 μL reaction volume, with each tube containing a 1:10 dilution of total converted cDNA, and 1 μm final primer concentration for each forward and reverse primer. PCR was performed as follows: 5 min at 95°C; 35 cycles of 5 s at 95°C and 10 s at 60°C; melt curve ramping from 60°C to 95°C, rising by 1°C at each step.

### Additional 2019 cases

Two additional TB foals with idiopathic hypocalcemia were diagnosed and subsequently genotyped after the discovery of the *RAPGEF5* nonsense variant. In April of 2019, a 2-day-old tachycardic and febrile (102.6°F) TB filly (*Case #3*) was admitted to Rhinebeck Equine L.L.P (Rhinebeck, NY) for acute respiratory distress of unknown cause. The foal was dull and recumbent at arrival, with a moderate suckle reflex, nasal flare and moderate peripheral pulse quality. Initial blood work revealed hypocalcemia (total calcium 6.6 mg/dL; ionized calcium 0.8 mmol/L) and hypoalbuminemia (2.6 g/dL [ref: 3.0–4.0 g/dL]). The filly was started on intravenous fluids containing calcium gluconate and antimicrobials were administered. The filly became brighter with treatment and was able to stand with no assistance. Subsequent blood worked revealed refractory hypocalcemia (total 7.9 mg/dL, ionized calcium 0.9 mmol/L). PTH concentrations (1.2 pmol/L) were inappropriately normal. The foal remained hypocalcemic despite maintenance on IV calcium gluconate, in addition to oral calcium carbonate supplementation mic. The filly was euthanized due to poor prognosis and a complete postmortem examination was performed.

A one-hour-old TB colt (*Case #4*) was presented to the University of Pennsylvania’s New Bolton Center (NBC, Kennett Square, PA) on April 2019 for a history of dark fetal fluids and apnea following dystocia. The colt stopped breathing despite being on supplemental oxygen following delivery. The colt’s vitals, respiratory pattern, and effort reached normal limits after caffeine administration; however, the foal was unable to stand without assistance and had bilateral moderate subscleral hemorrhage. Antimicrobial therapy was administered due to concerns for possible meconium aspiration. Initial blood work showed increased creatinine (2.6 mg/dL [ref: 0.6–1.8 mg/dL]), creatine kinase (4101 U/L; 90–270 U/L), and total bilirubin (2.7 mg/dL), in addition to hypophosphatemia (4.30 g/dL [ref: 4.60–6.90 g/dL]), hypoalbuminemia (2.4 g/dL) and very mild total hypocalcemia (10.72 mg/dL; ionized calcium 6.21 mg/dL). The foal was discharged on antimicrobial therapy.

At six-weeks of age, the colt was re-admitted to NBC for evaluation and treatment of seizures and hypocalcemic tetany. Diagnostics performed on the farm revealed marked hypocalcemia (total calcium 4.7 mg/dL), hypomagnesemia (1.3 mg/dL), hyperphosphatemia (14.1 mg/dL) and inappropriately normal PTH concentrations (5.9 pmol/L). At presentation, the colt was febrile (102.3°F), appeared stiff and displayed muscle fasciculations. An intravenous catheter was placed to administer calcium supplementation. Additionally, the foal was administered three grams of calcium carbonate orally every six hours. Subsequent blood work revealed persistent hypocalcemia (total calcium 5.27 mg/dL; ionized calcium 2.64 mg/dL, hypomagnesemia 0.9 mg/dL and hyperphosphatemia 14.95 mg/dL). The foal was maintained on IV and oral calcium supplementation. For seizure control, multiple centrally acting anti-seizure medications including benzodiazepines, barbiturates, and propofol were administered. Unfortunately, the foal continued to display seizure activity. Due to the poor prognosis for recovery, the colt was euthanized, and a full necropsy was performed.

### *RAPGEF5* c.2624C>A p.Ser875* functional validation

The *RAPGEF5* nonsense mutation leads to a cytosine-to-adenine change, resulting in a serine amino acid to change into a premature stop codon, possibly truncating an essential domain to the protein’s amino acid sequence. Therefore, we hypothesized that protein function would be impaired.

### Xenopus

*Xenopus tropicalis* were housed and cared for in the aquatics facility following Yale University Institutional Animal Care and Use Committee (IACUC) protocols and all methods carried out in accordance with this protocol. Embryos were produced and raised as previously described [30].

### mRNA generation

Custom synthesis of both wildtype and mutant (p.Ser875*) equine *RAPGEF5* cDNA, including untranslated regions (UTRs), was performed by GeneWiz (South Plainfield, NJ) with sequence verification and custom cloning into pCS108 (ampicillin) via 5’ BamHI and 3’Xhol for expression in *Xenopus*. We generated mRNA *in vitro* using mMessage machine kits (Ambion) from these plasmid templates by following the manufacturer’s instructions. Full cDNA sequences are available in **S4 Fig**.

### Embryo injection and phenotyping

Female *Xenopus tropicalis* were induced to ovulate and *in vitro* fertilization performed according to a standard protocol [31]. Fertilized embryos were injected with 200pg/embryo of either wild type equine *RAPGEF5* mRNA, equine S875* *RAPGEF5* mRNA or GFP at the one-cell stage. Embryos were incubated at 25°C for ~25 hours and developed to stage 28 (per Nieuwkoop and Faber [32]). At this stage, embryos were scored for developmental defects. Uninjected controls (n = 401), equine *RAPGEF5* injected (n = 313), equine S875* *RAPGEF5* (n = 218) injected and GFP injected (n = 260) embryos were sorted into four categories of developmental defects: normal, mild, moderate, and severe. Mildly affected embryos completed gastrulation and neurulation but did not elongate completely and had abnormal tails. Moderately affected embryos failed to form head structures although tail structures were present. Severely affected embryos completely failed gastrulation with open neural tubes and no evidence of head structures such as the eye and lacked tail elongation.

### Statistical analysis

All *Xenopus* experiments were performed a minimum of three times and results are presented as the composite of multiple experiments. Statistical significance of abnormalities was tested using Fisher’s exact tests with significance set at *P*<0.05.

## Supporting information

S1 Fig Extended pedigree of the four foals affected with idiopathic hypocalcemia. (PNG)

S2 Fig Protein sequence alignment of the X1 and X2 equine protein isoforms of RAPGEF5 closer to the C terminus, demonstrating a single glutamine deletion (black box) at p.331. Note that alignments are across species and the amino acid location bar is based on alignment and not the equine amino acid sequence.
(PNG)

S3 Fig Protein structure of human RAPGEF5, with the altered serine residue highlighted by a red box. This serine residue was associated with a putative phosphorylation site.
(PDF)

S4 Fig FASTA files of the full-length wildtype and mutant equine *RAPGEF5* cDNA used to create constructs. (TXT)

S1 Table Regions of homozygosity identified across two affected foals using whole-genome sequence data pruned for linkage disequilibrium. (XLSX)

S2 Table vcf files for significantly associated genetic variants within regions of homozygosity. ‘HIGH’ effects variants are highlighted in yellow and ‘MODERATE’ effects variants are highlighted in blue.
(XLSX)

S3 Table Healthy subset of Thoroughbreds used to determine an allele frequency for the *RAPGEF5* nonsense variant. (XLSX)

S4 Table mRNA alignment of equine isoform X1 predicted RAPGEF5 (XM_023639352.1) to equine isoform X2 (XM_023639353.1) and five validated human RAPGEF5 mRNA sequences. (XLSX)
