# Supplementary figures and images for: A nonsense variant in Rap Guanine Nucleotide Exchange Factor 5 (RAPGEF5) is associated with equine familial isolated hypoparathyroidism in Thoroughbred foals
Source: PLoS Genet. 2020 Sep 28;16(9):e1009028. doi: 10.1371/journal.pgen.1009028 (PMC7544121; doi:10.1371/journal.pgen.1009028)

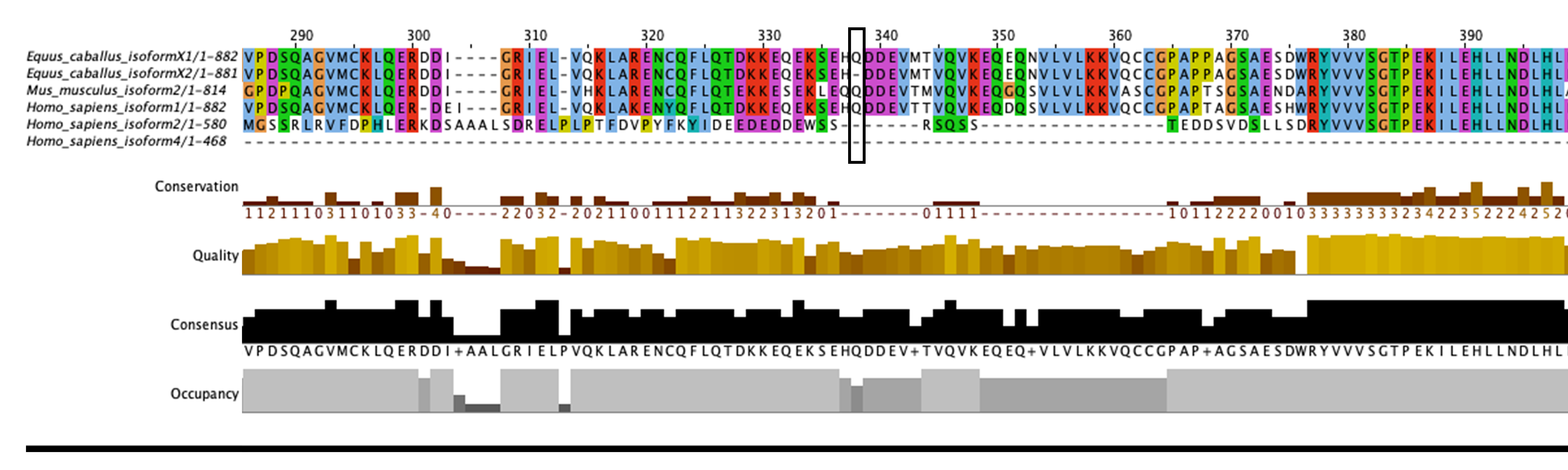

Supplement: S2 Fig — Note that alignments are across species and the amino acid location bar is based on alignment and not the equine amino acid sequence. (PNG) [file pgen.1009028.s002.png]

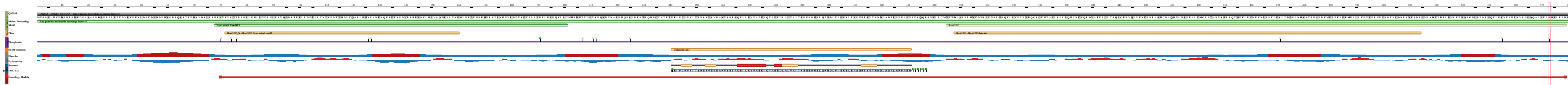

Supplement: S3 Fig — This serine residue was associated with a putative phosphorylation site. (PDF) [file pgen.1009028.s003.pdf]
